# Two lives, one bite: a six-year retrospective study on snakebite envenoming among pregnant women in Northeastern Nigeria

**DOI:** 10.3389/fgwh.2025.1655068

**Published:** 2025-09-17

**Authors:** Nicholas Amani Hamman, Aashna Uppal, Nuhu Mohammed, Nyadah Nicholas, Abubakar Saidu Ballah, Mohammed Bello Seyoji, Danimoh Mustapha Abdulsalam, Mohammed Buwa Garba, Joshua Abubakar Difa, Arancha De La Horra

**Affiliations:** 1 Snakebite Treatment and Research Hospital, Kaltungo, Nigeria; 2 The Global Health Network, Centre for Global Health and Tropical Medicine, University of Oxford, Oxford, United Kingdom; 3 Gombe State Hospital Services Management Board, Gombe, Nigeria; 4 Department of Community Medicine, Gombe State University/Federal Teaching Hospital Gombe, Gombe, Nigeria; 5 Department of Obstetrics and Gynecology, Gombe State University/State Specialist Hospital, Gombe, Nigeria

**Keywords:** snakebite, pregnancy, outcomes, epidemiology, Nigeria

## Abstract

**Introduction:**

Pregnant women with neglected tropical diseases like snakebites are considered doubly neglected due to the intersection of multiple vulnerabilities, including compounded challenges stemming from socio-economic marginalization, inadequate healthcare access and most importantly, the lack of targeted public health interventions. Despite these concerns, there is a substantial gap in the literature regarding the presentation, management and outcomes of snakebites among pregnant women, especially in low-resource settings like northeastern Nigeria.

**Methods:**

Consequently, a retrospective observational study was conducted at the Snakebite Treatment and Research Hospital (SBTRH) in Kaltungo, Northeastern Nigeria. Six years of patient folders were searched to identify patients of all ages that were pregnant at the time of presentation.

**Results:**

Between 2019 and 2024, 77 pregnant women presented to SBTRH with snakebites. The median age among pregnant women presenting with snakebite was 26 [interquartile range (IQR) 20–30], and most had not completed any level of education (*n* = 70, 91%). On average, patients were not experiencing their first pregnancy (median gravidity 3, IQR 1–5), and the median gestational age at admission was 22 weeks (IQR 16–28 weeks). Most patients (*n* = 73, 95%) visited a traditional healer prior to arriving at SBTRH. All patients recovered from snakebite. Of the two women that gave birth prior to discharge, one underwent spontaneous vaginal delivery followed by neonatal death, and one underwent caesarean section, where both mother and child survived.

**Discussion:**

These findings underscore the urgent need to recognize and respond to the unique vulnerabilities of pregnant women affected by snakebite in northeastern Nigeria. There is a need to integrate snakebite education during antenatal care period, engaging traditional healers in referral networks, developing pregnancy-specific clinical protocols and strengthening surveillance systems to capture maternal and foetal outcomes comprehensively.

## 1 Introduction

Snakebite envenoming is a very serious public health issue that remains underappreciated despite reported high mortality and morbidity, especially in rural and underserved communities of Asia, sub-Saharan Africa and parts of Latin America (1–3). The World Health Organization (WHO) has recognized snakebite envenoming as a neglected tropical disease (NTD), highlighting its high burden particularly in rural areas where access to health care is very limited (4). WHO estimates that between 81,000 and 138,000 people die annually from snakebites worldwide with approximately three times that number surviving but suffering from amputations and permanent disabilities (5, 6). However, the WHO aims to halve these deaths and disabilities by 2030, through ensuring access to safe, affordable treatment, empowering communities, strengthening health systems and building a global coalition for advocacy, resources mobilization and effective implementation of management victims of snake bite (6). Despite the WHO's efforts to address the burden of snakebite envenoming globally, many regions, particularly in sub-Saharan Africa, continue to face significant challenges in managing this condition effectively (7). The challenge arises from the demographics of those mostly affected by snakebites—marginalized and vulnerable populations, often from rural or impoverished areas, who face limited access to healthcare and with limited political and societal influence, thereby leading to their needs being overlooked in public health policies and interventions (6).

In Nigeria, the story is similar, with a study in 2019 estimating that Nigeria experiences 43,049 snakebite cases annually, resulting in 1,927 deaths and 2,368 amputations, with the most vulnerable populations being farmers, herdsmen and their rural families (8). This population group contributes significantly to the economy and their snakebite-related incapacitation leads to reduced agricultural productivity and economic losses (3). This challenge is exacerbated by lack of adequate healthcare infrastructure, particularly in rural areas, resulting in limited access to timely and effective antivenom (9).

In many snakebite-prone regions of the world, including northeastern Nigeria, men are disproportionately affected due to their involvement in outdoor farming activities (10–12). Recent epidemiological data from northeastern Nigeria have also highlighted the considerable burden among children and adolescents (13). Importantly, it is pertinent to note that pregnant women and other women of child-bearing age are also exposed to various environmental and occupational activities such as firewood collection, farming, and water fetching that increase their vulnerabilities to snakebites (14). Pregnant women with snakebite envenoming are considered doubly neglected due to the intersection of multiple vulnerabilities, including compounded challenges stemming from socio-economic marginalization, inadequate healthcare access and most importantly, the lack of targeted public health interventions, all of which may impair maternal and foetal health (15).

The physiological and immunological changes that occur during pregnancy can significantly alter the body's response external stressors, making pregnant women more vulnerable to the complications following snakebites (14, 16). These changes may also affect the metabolism and pathophysiological effects of venom, leading to potentially severe and multifaceted complications in pregnant women (17). For instance, haematoxic snake venom, which is prevalent in Nigeria (8), may cause systemic coagulopathy. When this effect is combined with the already altered coagulation status of the pregnant body, it may lead to vaginal bleeding, hemorrhagic and other life-threatening complications (17, 18). Moreover, while anti-venom remains the mainstay of snakebite treatment and has not been associated with adverse maternal or foetal outcomes, there is limited evidence to guide optimal dosing specifically in pregnant women (17, 18). The fact that the same dose is being administered for both pregnant and non-pregnant individuals continue to raise concern regarding its potential risks and impacts on maternal and foetal outcomes (17). Compounding these medical challenges in northeastern Nigeria is the widespread reliance on traditional medicine for snakebite treatment. Many rural communities have deep-rooted beliefs in traditional healing practices, leading victims to seek care from traditional healers before accessing formal healthcare, often resulting in critical delays in receiving appropriate medical intervention (19).

Despite these concerns, there is a substantial gap in the literature regarding the presentation, management and outcomes of snakebites among pregnant women, especially in low-resource settings like northeastern Nigeria. Recommendations for the management of envenomation in pregnancy are guided primarily by studies on non-pregnant individuals and children, with minimal consideration for the unique needs of pregnant women, who may require specialized care and treatment due to the potential risk to both the mother and the foetus. This lack of information creates a critical gap in understanding the specific challenges and best practices for managing snakebite incidents in this vulnerable group. Accordingly, this study aims to investigate the presentation, management and outcomes of snakebite in pregnant women in Northeastern Nigeria over a six-year period (2019–2024) using retrospective data. Particular attention is given to identifying the unique challenges faced in low-resource settings. The findings of this study will help address the current gap in the literature and inform health care strategies aimed at improving outcomes for both mothers and their unborn child.

## 2 Materials and methods

### 2.1 Study setting

A retrospective observational study was conducted at the Snakebite Treatment and Research Hospital (SBTRH) in Kaltungo, Gombe State, Northeastern Nigeria. This hospital is the largest of its kind in terms of patient load, with around 2,500 snakebite patients presenting annually from Gombe State, neighboring states, and patients also come from neighboring countries. SBTRH is dedicated to clinical management of snakebite envenoming, research, training, and public awareness campaigns to reduce the impact of snakebite morbidity and mortality in the region.

### 2.2 Patient inclusion criteria

A six-year review of patient records (2019–2024) was conducted to identify all pregnant patients presenting during this period. Following identification of relevant patient folders, a structured digital proforma was created using Kobo Toolbox, an open-source digital platform, to systematically collect data across four key domains: Patient's demographics (age, sex, state of origin, education level, ethnicity, occupation), Clinical characteristics (body mass index, blood pressure at admission), Snakebite -related details such as (date of bite, location where bite occurred, hours taken to arrive at hospital, anatomic site of bite, use of traditional medicine, snake species involved, antivenom dosage administered, number of blood transfusions required, final outcome following snakebite), and Pregnancy-specific characteristics (gravidity, gestational age at admission, vaginal bleeding, pregnancy outcome). Snake species identification was primarily determined at the time of patient presentation. In more than 50% of cases, patients brought the dead snake, which enabled accurate identification. When the snake was not available, photographs of common species displayed in the emergency unit were used to assist with identification. Cases in which the patient was unable to identify the snake were documented as “unidentified.”

### 2.3 Analysis

After data collection with Kobo Toolbox, the dataset was exported as a CSV file. Data was cleaned and analysed using R software (version 4.3.1). Descriptive statistics were employed to summarize patient characteristics. Additional analyses were conducted to explore potential associations between variables of interest. Specifically, Wilcoxon Rank Sum tests were used to compare the difference in hospital arrival times among patients who sought traditional medicine vs. those who did not, as well as the difference in gravidity among patients who experienced vaginal bleeding.

### 2.4 Ethical clearance

Ethical clearance was obtained from the Research and Ethics Committee, Gombe State Hospitals Services Management Board with reference number GS/HSMB/RES/S/05/VOL.75.

## 3 Results

**Figure 1:**
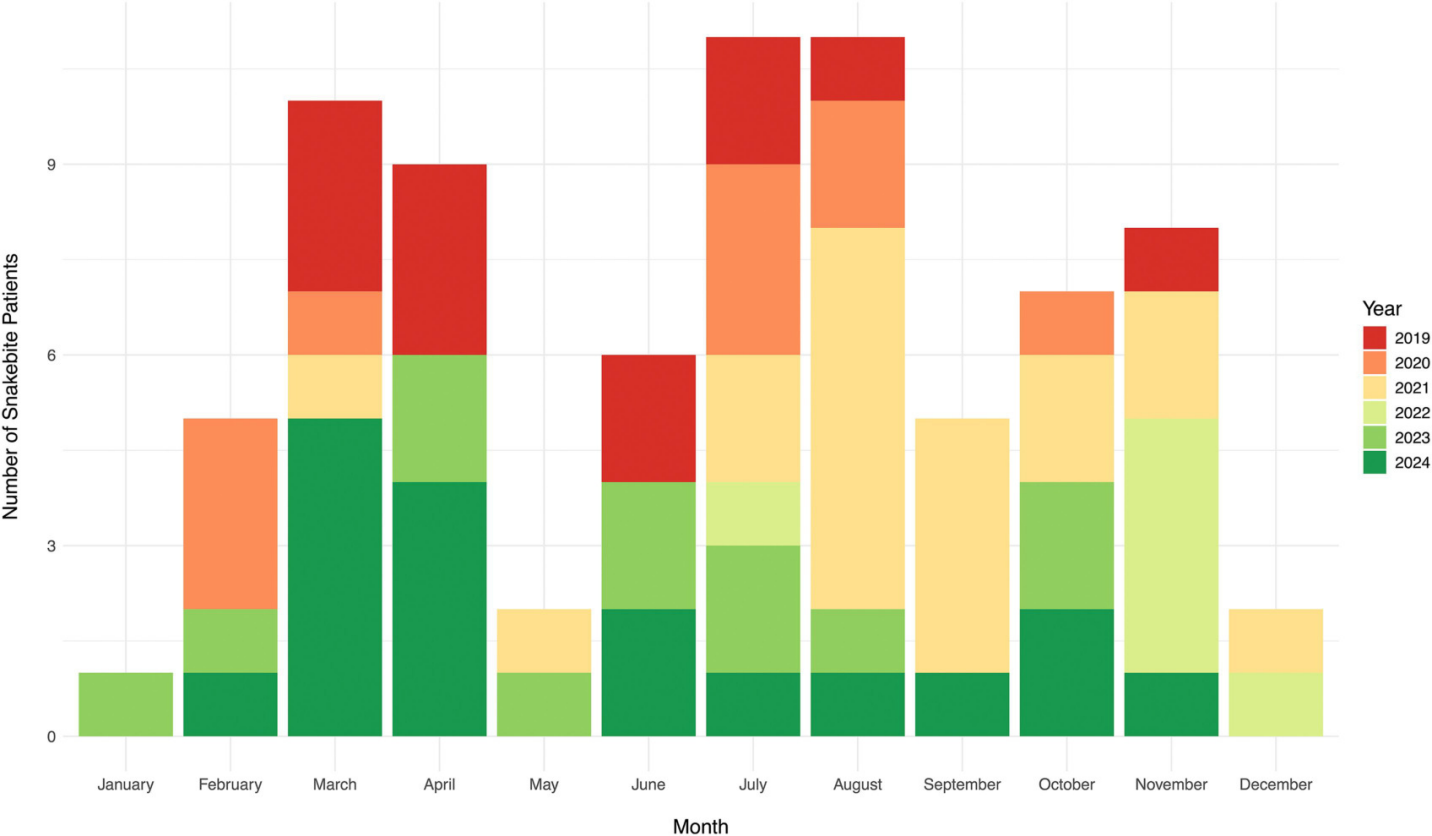
Number of pregnant women presenting with snakebites per month (2019–2024).

Between 2019 and 2024, a total of 77 pregnant women presented to SBTRH for snakebite. The number of cases varied annually, with the highest number of cases recorded in 2024 (*n* = 18, accounting for 23% of all cases) and the lowest in 2022 (*n* = 6, 8%). Snakebites among pregnant women also exhibited a seasonal trend. On average, peaks in the number of snakebite patients were observed in July (*n* = 11, 14%) and August (*n* = 11, 14%), followed by March (*n* = 10, 13%) (Figure 1).

Table 1 outlines additional patient characteristics. The median age of pregnant women presenting with snakebite was 26 [interquartile range (IQR) 20–30], and most had not completed any level of education (*n* = 70, 91%). There was diversity in patients’ state of origin and ethnicity. The majority of patients were from Gombe State (*n* = 31, 40%). Although there were 22 unique ethnicities represented among the cohort of patients, more than half of the patients identified as Fulani (*n* = 45, 58%). Further, most patients were housewives (*n* = 60, 81%). Clinical characteristics were within the normal range among the 54 patients for which this information was reported; the median body mass index was 22.6 (IQR 21.0–23.6), and median blood pressure was 120/79 (IQR 111/70–121/80).

**Table 1:** Patient demographics, clinical characteristics, and snakebite characteristics.

Characteristic	*N* = 77^a^
Demographics	
Age	26 (20, 30)
Highest level of education	
None	70 (91%)
Tertiary	5 (6.5%)
Secondary	2 (2.6%)
State of origin	
Gombe	31 (40%)
Adamawa	16 (21%)
Bauchi	11 (14%)
Taraba	10 (13%)
Borno	5 (6.4%)
Yobe	3 (3.9%)
Jigawa	1 (1.3%)
Ethnicity (Top 5)^b^	
Fulani	45 (58%)
Hausa	4 (5.2%)
Tangale	4 (5.2%)
Waja	4 (5.2%)
Tula	2 (2.6%)
Occupation	
Housewife	60 (81%)
Farmer	12 (16%)
Student	1 (1.4%)
Under care	1 (1.4%)
*Not reported*	*3* (*3.9%)*
Clinical characteristics	
Body mass index	22.6 (21.0, 23.6)
*Not reported*	*23*
Blood pressure at admission	120/79 (111/70, 121/80)
*Not reported*	*23*
Characteristics of snakebite	
Snake species	
Carpet viper	49 (64%)
Unidentifiable	23 (30%)
Cobra	5 (6.5%)
Time to hospital admission	1.00 (0.21, 2.00)
Site of bite	
Lower limb	64 (83%)
Upper limb	12 (16%)
Chest	1 (1.3%)
Place of bite occurrence	
Home	29 (38%)
Compound	27 (35%)
Farm	20 (26%)
Other	1 (1.3%)
Swelling at presentation	
Swelling	75 (97%)
No swelling	2 (2.6%)
Antivenom sose (number of vials)	1 (1, 2)
Number of blood transfusions	2 (1, 2)
*Not reported*	*45*
Snakebite outcome	
Recovery	77 (100%)

a Median (IQR); *n* (%).

b There are 22 unique ethnicities represented in this cohort of patients.

### 3.1 Snakebite characteristics

Nearly two-thirds of patients were bitten by Carpet Vipers (*n* = 49, 64%), with median time to hospital arrival of one hour (IQR 0.21–2 h). Most bites occurred on the lower limbs (*n* = 64, 83%), primarily at home (*n* = 29, 38%), within patients’ compounds (*n* = 27, 35%), or on farmland (*n* = 20, 26%). Nearly all the patients presented with swelling at or near the bite site (*n* = 75, 97%). All patients received at least one dose of antivenom, with a median of one dose administered (IQR 1–2). Of the 32 patients for whom blood transfusion information was available, the median number of blood transfusions was two (IQR 1–2). All patients recovered from snakebite following treatment, with no fatalities recorded.

### 3.2 Traditional medicine

The majority of patients (*n* = 73, 95%) sought care from a traditional healer before arriving at SBTRH, reflecting a common practice in this region. Of these, most received a combination of oral and topical remedies (*n* = 69, 96%) (Table 2). Notably, the median time to hospital arrival was longer among patients who consulted traditional healers (median 1 h, IQR 13 min–2 h), compared to those who came directly to SBTRH (median 11 min, IQR 4–28 min), however, this difference was not statistically significant (*p* value = 0.08).

**Table 2:** Use of traditional medicine.

Characteristic	*N* = 77^a^
Treated with traditional medicine prior to arrival	73 (95%)
Type of traditional medicine used	
Oral and topical	69 (96%)
Topical	2 (2.8%)
Oral	1 (1.4%)
Not reported	5

a Median (IQR); *n* (%).

### 3.3 Pregnancy characteristics

Table 3 outlines key pregnancy-related characteristics. On average, patients were on their third pregnancy (median gravidity 3, IQR 1–5). The median gestational age at admission was 22 weeks (IQR 16–28 weeks). One out of four patients presented with vaginal bleeding at admission (*n* = 19). Among those with vaginal bleeding, the median gravidity was 3 (IQR: 1–4), compared to a median of 3 (IQR: 1.5–5.5) among those without vaginal bleeding; this difference was not statistically significant (*p* = 0.2). Furthermore, the majority of the patients were still pregnant at the time of discharge (*n* = 75, 97%). Of the two women that gave birth prior to discharge, one underwent spontaneous vaginal delivery followed by neonatal death, and one underwent caesarean section, where both mother and child survived.

**Table 3:** Pregnancy characteristics.

Characteristic	*N* = 77^a^
Gravidity	3 (1, 5)
Not reported	1
Gestational age at admission (Weeks)	22 (16, 28)
Not reported	1
Vaginal bleeding at admission	19 (25%)
Pregnancy outcome at discharge	
Still pregnant at time of discharge	75 (97%)
Already given birth prior to time of discharge	2 (2.6%)

a Median (IQR); *n* (%).

## 4 Discussion

This study represents one of the few and, to our knowledge, the largest retrospective investigations of snakebite during pregnancy in sub-Saharan Africa, contributing valuable insights to a significantly under-researched issue. It provides insight into the demographic characteristics, clinical progression, and outcomes of pregnant women envenomed by snakebite in a high-burden setting, highlighting critical gaps in care and underscoring the urgent need for targeted interventions to improve maternal and foetal health outcomes.

Although snakebite in pregnancy remains relatively unrecognized in global maternal health discourse, its incidence in endemic regions may be comparable to that of other well-established obstetric emergencies. For instance, studies have reported prevalence rates of up to 0.9 per 1,000 pregnancies (0.09%) in parts of sub-Saharan Africa (20), approaching the global (21) and African (22) incidence of ectopic pregnancy, which occurs in approximately 1%–2% of pregnancies. While ectopic pregnancy is a well-documented gynecological emergency that primarily threatens maternal life, snakebite during pregnancy on the other hand endangers both maternal and foetal outcomes. Despite this dual burden, snakebite remains largely absent from reproductive and maternal health protocols. These findings emphasize the importance of integrating snakebite into clinical guidelines and research agendas, particularly in regions where the risk of envenoming coincides with high maternal vulnerability. Doing so could significantly enhance early recognition, appropriate management, and ultimately, improved outcomes for affected women and their unborn children.

The observed seasonal clustering of cases during the rainy months of July and August aligns with well-documented patterns of increased snakebite incidence during farming periods in endemic regions. During these times, heightened human activity in agricultural areas leads to greater overlap with snake habitats, increasing exposure risk across populations (1, 23–26). Although this trend has been widely reported in general epidemiological studies, its relevance to pregnant women remains understudied. However, the observed trend suggests that pregnant women in rural and agricultural communities may face heightened risk during certain times of the year, particularly during peak farming seasons, when increased agricultural activity leads to greater overlap between human and snake habitat. This finding has important public health implications, highlighting the need for targeted prevention strategies. Community education on preventive measures such as wearing protective footwear, avoiding high-risk areas and seeking timely medical care should be prioritized particularly during high-risk months. Furthermore, antenatal care programs in endemic regions could incorporate seasonal risk awareness and snakebite first-aid education to better safeguard this vulnerable population.

In addition, the majority of patients in this study faced significant socioeconomic disadvantages, with 91% lacking formal education and 81% identifying as housewives. These factors are commonly associated with delayed healthcare-seeking behavior and poorer outcomes, particularly in the context of pregnancy-related complications (27–29) and snakebite envenomation (30–32). Notably, 95% of patients first sought care from traditional healers prior to hospital presentation, reflecting the strong cultural reliance on traditional medicine in rural settings, a common occurrence in cases of snakebite envenoming (33, 34). Although the difference in arrival time to hospital presentation between those who did and did not consult traditional healers was not statistically significant (*p* = 0.08), even small delays may have clinical implications; the lack of statistical significance may also reflect the study's limited sample size and should not dismiss potential clinical risks. Consulting traditional healers for incident of snakebite is common practice in this region, and often reflects patients’ first point of care before proceeding to more definitive care in the hospital. This concern is heightened in pregnant patients, where treatment delays can have serious consequences for both maternal and foetal outcomes (14). In our study, the median time to hospital arrival was relatively short compared to other reports (median 1 h for those consulting traditional healers vs. 11 min for those presenting directly). Given that only 40% of patients were from Gombe State, this finding cannot be explained solely by geographical proximity. The reasons remain uncertain and may involve factors such as referral patterns, transport availability, or increased awareness of hospital services among pregnant women. However, our data did not directly assess these aspects, and further research is needed to clarify the determinants of timely presentation. Nonetheless, these findings underscore the need for multifaceted interventions that address both cultural and educational barriers. Strategies such as engaging traditional healers in public health initiatives and implementing literacy programs tailored to women may empower communities to make timely and informed healthcare decisions ultimately improving health outcomes in vulnerable populations.

Bite location and context revealed a crucial insight as most bites (73%) occurred not in occupational farmland or bush settings but within domestic and peri-domestic spaces. This challenges dominant narratives that associate snakebite risk solely with farm, herding or walking in the bush path (14, 35–37) and suggests that even housebound women are not spared. In rural northern Nigeria, open compounds, thatched floors, and firewood storage areas within living quarters present daily exposure risks. Local governments should incorporate low-cost environmental interventions such as community-driven compound cleaning, safe firewood storage and housing improvement schemes into their broader maternal and child health programs. These preventive measures, though simple, can significantly reduce the environmental risk of bites particularly in settings where avoidance of outdoor labour does not ensure safety.

The predominance of carpet viper (*Echis ocellatus)* bites (64%) confirms the expected pattern of envenoming in this region where haematoxic snake species are most common (14, 35). Indeed, carpet viper remains the most common species implicated in snakebite envenoming in this region, as noted in our previous work (12). The presence of vaginal bleeding in one-quarter of patients is clinically concerning and highlights the hematotoxic nature of Viperidae envenomation (38). This necessitates high clinical suspicion for obstetric complications upon presentation even in the absence of overt symptoms. A recommendation here is for clinicians in snakebite treatment centers to integrate routine obstetric consultation in all pregnant cases and to develop a modified clinical protocol for obstetric monitoring including early ultrasound and coagulation profiling when resources permit. These simple yet targeted adjustments could enable earlier detection of foetal compromise and guide timely intervention. Although no statistically significant relationship was found between gravidity and bleeding, the clinical severity observed in some cases underscores the need for more detailed analyses of factors such as obstetric history and time to treatment. These variables have not been fully examined in this retrospective study due to data availability, representing important areas for future research. Given the number of unreported parameters in our dataset and the severe consequences of snakebite during pregnancy, we recommend targeted measures to improve documentation. Accurate and consistent recording of key demographic and clinical information should be prioritized not only within our facility but also across other hospitals managing snakebite cases. Documentation practices should be tailored to the specifics for pregnant women. To support this, a simplified, low-burden tick-box system can be incorporated into patient folders or case notes, enabling essential data capture without overburdening frontline healthcare providers. Additionally, future studies should stratify patients by gestational age and trimester to assess how timing of envenoming influences pregnancy outcomes. Establishing centralized snakebite registries that incorporate pregnancy-specific data would enable more accurate and evidence-based public health planning moving beyond generalized assumptions drawn from non-pregnant populations.

Maternal outcomes in this study were overwhelmingly positive with all 77 patients surviving and 97% remaining pregnant at discharge. This aligns with findings from the only comparable study conducted at the same facility over 17 years ago (20). However, the single neonatal death following spontaneous delivery highlights a broader gap in snakebite outcome metrics, where foetal and neonatal outcomes are often overlooked. While maternal outcomes were positive, detailed foetal outcomes such as birth weight, Apgar score, congenital anomalies or longer neonatal health were not captured in this study. Future prospective studies should consider perinatal outcome tracking through follow-up mechanisms or integration with maternal and child health records. These two births, though limited in number, highlight the variability in maternal-foetal response to snakebite and the need for individualized management pathways based on obstetric risk assessment. Health records and surveillance systems should be adapted to include standardized documentation of foetal well-being, neonatal status and post-discharge follow-up for pregnant snakebite victims. Recommended measures include structured obstetric monitoring after discharge and psychosocial support services tailored to pregnancy-related complications of envenoming, including perinatal loss.

While antivenom remains the cornerstone of snakebite treatment (20) and was administered to all patients without observed adverse effects, concerns persist about the uniformity of dosing in pregnancy. Of note, patients in this setting receive Echitab ICP polyvalent antivenom, which was locally developed in Nigeria with production in Costa Rica. This brand has been shown to be more effective than PANAF-Premium in the Nigerian context, which explains the lower dosage in our institution (1–3 vials) (39). Our findings are consistent with previous studies, showing that most patients treated with Echitab recover with a single vial (12, 39). Further, the study did not report any adverse reactions to antivenom; however, the retrospective nature of our data and potential under reporting limit the ability to confirm their absence. The altered pharmacokinetics of pregnancy such as increased plasma volume and renal filtration (40) raise important questions about efficacy, optimal timing, and potential under-dosing or overexposure. Although this study did not observe any adverse effects, it cannot rule out subtle or delayed consequences on foetal development or maternal recovery. There is a strong case here for investment in pharmacokinetic and safety studies of antivenom use in pregnancy, which would enable the creation of trimester-specific dosing recommendations and strengthen clinician confidence in managing this unique population. Until such evidence emerges, a cautious yet proactive approach involving multidisciplinary teams and close foetal surveillance should guide clinical decision-making.

The overarching silence around pregnant snakebite victims in policy documents, training curricula and surveillance reports remains deeply troubling. This study not only highlights the biological and clinical challenges involved but also unveils the systemic neglect that shapes poor health outcomes. Pregnant women in envenoming-endemic zones are trapped within overlapping spheres of invisibility—medically underserved, socially marginalized, and largely excluded from strategic interventions in global and national health planning. The relative absence of their stories from both research and policy reflects a larger issue of whose lives are deemed visible enough to matter in public health priorities. Snakebite policies must evolve to recognize pregnancy as a distinct clinical and public health concern, deserving of dedicated protocols, targeted research funding and culturally responsive community outreach strategies.

This study, while offering important clinical insights is limited by its retrospective design which constrained the ability to control for confounding variables or assess temporal relationships between envenoming and obstetric outcomes. The absence of long-term maternal and neonatal follow-up limits the evaluation of delayed complications or developmental impacts. Additionally, data on critical variables such as obstetric history, time to antivenom administration and pharmacokinetic changes during pregnancy were either unavailable or inconsistently documented, restricting the depth of analysis. The severity of envenoming was not formally classified, though indirect indicators such as the number of antivenom vials and blood transfusion were available which may serve as proxies in future studies to estimate case severity. Although the study was conducted at a single center, it treats patients from all states in northeastern Nigeria as well as neighboring countries, which partially addresses concerns about generalizability. However, regional differences in healthcare systems, cultural practices and patient demographics could still limit the applicability of the findings to other areas or healthcare settings. Despite these constraints, the findings provide a compelling case for more robust, prospective, and multi-center research to inform pregnancy-specific protocols and public health responses.

Indeed, this study underscores the urgent need to recognize and respond to the unique vulnerabilities of pregnant women affected by snakebite in northeastern Nigeria. The intersection of socioeconomic marginalization, delayed health-seeking behavior and physiological susceptibility creates a perfect storm of risk. While maternal survival in this cohort was commendable, foetal outcomes and clinical complications highlight critical gaps in both care delivery and policy response. Recommendations emerging from this work include integrating snakebite education into antenatal care, engaging traditional healers in referral networks as potential collaborators, particularly for timely referral of snakebite patients to health facilities, developing pregnancy-specific clinical protocols and strengthening surveillance systems to capture maternal and foetal outcomes comprehensively in endemic regions. Ultimately, these interventions must be woven into a broader reimagining of maternal health in neglected tropical disease settings where invisibility can no longer be an acceptable diagnosis.

## Data Availability

The data analyzed in this study is subject to the following licenses/restrictions: data is held by the medical records team at the Snakebite Treatment and Research Hospital. However, the datasets analyzed for this study can be made available upon reasonable request to the corresponding authors. Requests to access these datasets should be directed to nicholas.amani4u@gmail.com.
